# *Shewanella* phage encoding a putative anti-CRISPR-like gene represents a novel potential viral family

**DOI:** 10.1128/spectrum.03367-23

**Published:** 2024-01-12

**Authors:** Hongmin Wang, Kaiyang Zheng, Min Wang, Keran Ma, Linyi Ren, Ruizhe Guo, Lina Ma, Hong Zhang, Yundan Liu, Yao Xiong, Miaolan Wu, Hongbing Shao, Yeong Yik Sung, Wen Jye Mok, Li Lian Wong, Andrew McMinn, Yantao Liang

**Affiliations:** 1 College of Marine Life Sciences, Institute of Evolution and Marine Biodiversity, MOE Key Laboratory of Evolution and Marine Biodiversity, Frontiers Science Center for Deep Ocean Multispheres and Earth System, Center for Ocean Carbon Neutrality, Ocean University of China, Qingdao, China; 2 Haide College, Ocean University of China, Qingdao, China; 3 Universiti Malaysia Terengganu-Ocean Unversity of China Joint Centre for Marine Studies, Qingdao, China; 4 The Affiliated Hospital of Qingdao University, Qingdao, China; 5 School of Life Sciences and Biotechnology, Shanghai Jiao Tong University, Shanghai, China; 6 Institute of Marine Biotechnology, Universiti Malaysia Terengganu, Kuala Terengganu, Malaysia; 7 Institute for Marine and Antarctic Studies, University of Tasmania, Hobart, Tasmania, Australia; USDA-ARS National Center for Cool and Cold Water Aquaculture, Kearneysville, West Virginia, USA

**Keywords:** *Shewanella *phage, genomic and comparative genomic analysis, phylogenetic analysis, anti-CRISPR AcrVA2, *Ranviridae*

## Abstract

*Shewanella* is a prevalent bacterial genus in deep-sea environments including marine sediments, exhibiting diverse metabolic capabilities that indicate its significant contributions to the marine biogeochemical cycles. However, only a few *Shewanella* phages were isolated and deposited in the NCBI database. In this study, we report the isolation and characterization of a novel *Shewanella* phage, vB_SbaS_Y11, that infects *Shewanella KR11* and was isolated from the sewage in Qingdao, China. Transmission electron microscopy revealed that vB_SbaS_Y11 has an icosahedral head and a long tail. The genome of vB_SbaS_Y11 is a linear, double-stranded DNA with a length of 62,799 bp and a G+C content of 46.9%, encoding 71 putative open reading frames. No tRNA genes or integrase-related feature genes were identified. An uncharacterized anti-CRISPR *AcrVA2* gene was detected in its genome. Phylogenetic analysis based on the amino acid sequences of whole genomes and comparative genomic analyses indicate that vB_SbaS_Y11 has a novel genomic architecture and shares low similarity to *Pseudomonas* virus H66 and *Pseudomonas* phage F116. vB_SbaS_Y11 represents a potential new family-level virus cluster with eight metagenomic assembled viral genomes named *Ranviridae*.

**IMPORTANCE:**

The Gram-negative *Shewanella* bacterial genus currently includes about 80 species of mostly aquatic *Gammaproteobacteria*, which were isolated around the globe in a multitude of environments, such as freshwater, seawater, coastal sediments, and the deepest trenches. Here, we present a *Shewanella* phage vB_SbaS_Y11 that contains an uncharacterized anti-CRISPR *AcrVA2* gene and belongs to a potential virus family, *Ranviridae*. This study will enhance the knowledge about the genome, diversity, taxonomic classification, and global distribution of *Shewanella* phage populations.

## INTRODUCTION

Viruses, as the most abundant biological entities, play critical roles in shaping microbial abundance and regulating the community structure (1, 2). They impact the nutrient cycle and energy flow in the ecosystems, thus regulating global biogeochemical cycles (2). Although metagenomic methods have revealed the vast genetic diversity of marine viruses, there is still a significant amount of unknown sequences called as “viral dark matter” (3). This is due to the lack of viral reference genomes in databases, particularly for ubiquitous phages that infect essential microbial clades across the ocean (3, 4). Therefore, isolating and characterizing novel phages are crucial to extending our understanding of their diversity, taxonomic status, and global distribution.

*Shewanella* is a facultative anaerobic bacterium belonging to the family *Vibrionaceae* in the order *Gammaproteobacteria*, known for its diverse respiratory capabilities, and is a model organism for studies on metal reduction and fuel cells (5–7). Some *Shewanella* are also potential pathogens of humans and aquatic animals, including *Shewanella putrefacient* and *Shewanella algae* (6, 8). In 1931, a bacterium was isolated from a contaminated Canadian butte (9). This strain was initially described as belonging to the genus *Achromobacter* and was reclassified half a century later into the newly created genus *Shewanella* (9). This genus currently includes about 80 species. Further investigation into the metabolic systems of *Shewanella* may provide a deeper comprehension of viral–host relationships in different ecological environments and offer valuable insights into the genetics and ecology of virus–host interactions. However, to date, only 21 *Shewanella*-infecting phages with a complete nucleotide genome have been reported on the NCBI virus database. This lags considerably behind studies of phages that infect other marine bacterial clades, such as *cyanophages*, *vibriophages*, and *roseophages* (10).

Many archaea and bacteria possess adaptive immune systems consisting of clustered regularly interspaced short palindromic repeats (CRISPR) and CRISPR-associated (Cas) proteins to defend against phage invasion (11, 12). In response, phages express anti-CRISPR (Acr) proteins to suppress CRISPR-dependent response (12). In 2018, a study reported five proteins (AcrVA1-5) that could inhibit the type V-A CRISPR-Cas12a system (13, 14). Among them, the mechanisms of action of AcrVA1, AcrVA4, and AcrVA5 have been well characterized. They inhibit Cas12a activity by degrading the crRNA of Cas12a, blocking the binding of Cas12a to target DNA, and acetylating the modification of Cas12a, respectively (11, 14–17). However, to date, no detailed studies have been reported on the structure and function of the other two AcrVA proteins (AcrVA2 and AcrVA3).

In this study, a novel vB_SbaS_Y11 encoding a putative anti-CRISPR AcrVA2 gene infecting *Shewanella KR11* was isolated from the sewage in Qingdao, China. Morphological, physiology experiment, genomic, phylogenetic, and biogeographic analyses of vB_SbaS_Y11 were undertaken. This study contributes to a better understanding of the genomic features, host interactions, and ecological roles of *Shewanella* phages.

## RESULTS AND DISCUSSION

### Morphology and characterization of vB_SbaS_Y11

**Fig 1:**
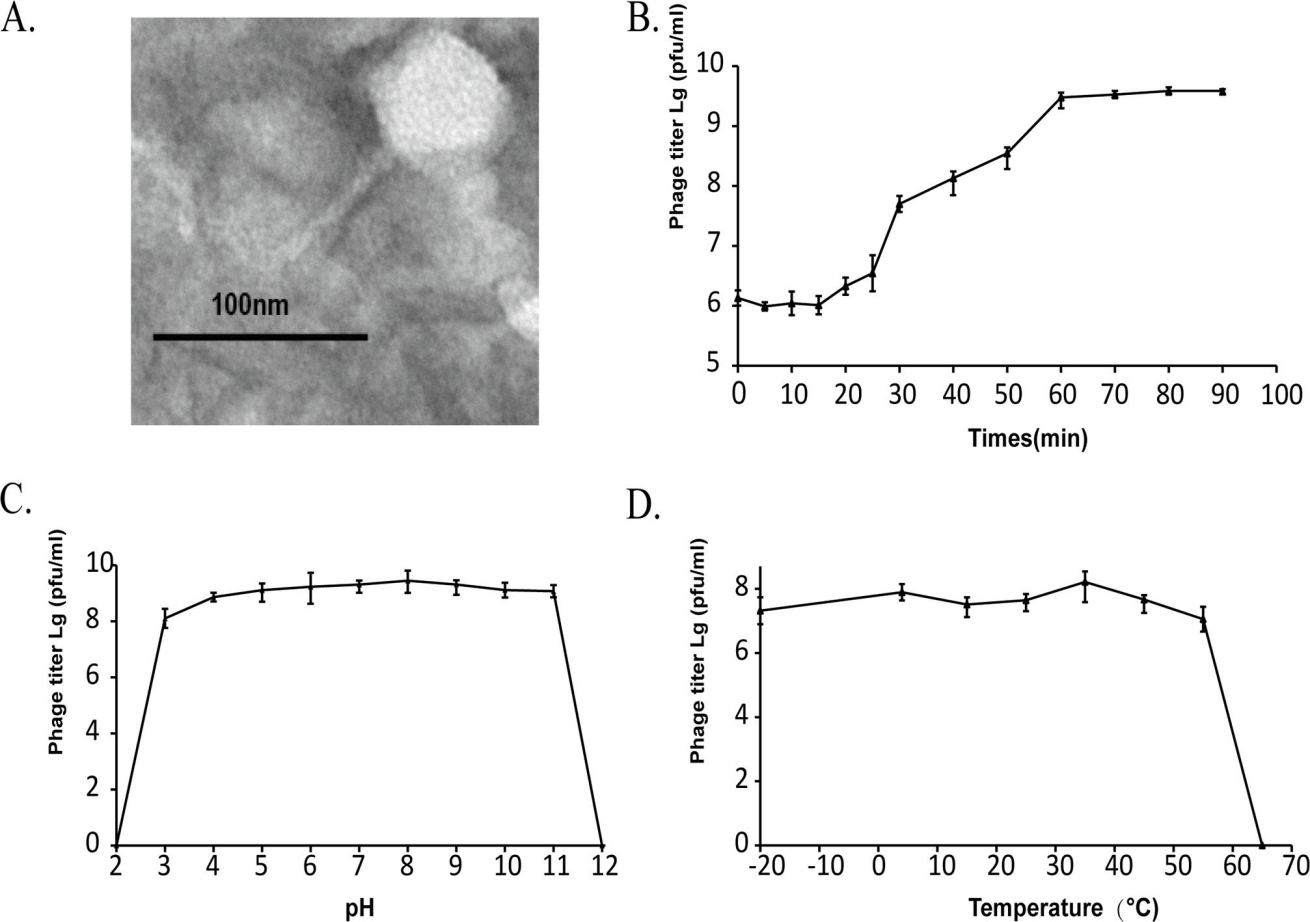
Biological properties of *Shewanella* phage vB_SbaS_Y11. (**A**) TEM morphology of *Shewanella* phage vB_SbaS_Y11. Phages were negatively stained with potassium phosphotungstate. Scale bar, 100 nm. (**B**) One-step growth curve of *Shewanella* phage vB_SbaS_Y11. (**C**) The curve of pH stability. (**D**) Thermal stability of *Shewanella* phage vB_SbaS_Y11.

The *Shewanella* phage vB_SbaS_Y11 was isolated from the sewage of a seafood market in Qingdao, China (36.063°N, 120.320°E), with *Shewanella KR11* as a host. Infection of vB_SbaS_Y11 formed clear circular patches of 2 mm in diameter in double-layer agar (Fig. S1). Transmission electron microscopy (TEM) images displayed that vB_SbaS_Y11 has a typical siphoviral morphology, containing an icosahedral head (average diameter about 57.8 ± 2.4) and a long noncontractile tail (average length about 72.2 ± 1.5) (Fig. 1A). Experimental results of viral one-step growth, temperature tolerance, and pH stability revealed the characteristics of vB_SbaS_Y11. The one-step growth curve analysis revealed that vB_SbaS_Y11 has a short latent period of approximately 15 minutes, followed by a rapid growth period of less than 45 minutes, and reaches a growth plateau after 60 minutes (Fig. 1B). In the temperature stability experiment, the phage exhibited maximum titers at approximately 35°C, with high and stable titers observed when the temperature ranged from −20°C to 55°C (Fig. 1D). However, the phage titer decreased linearly after 55°C, indicating that vB_SbaS_Y11 is intolerant to high temperatures. The possible reason for this is that in order to thrive in a water environment with lower average temperatures, these *Shewanella* bacteria should develop resistance mechanisms to achieve optimal growth at low temperatures (18). The activity of phage vB_SbaS_Y11 was found to be more stable between pH 4 and pH 10, with the highest titer observed at pH 8 (Fig. 1C). The phage titer sharply decreased when the pH exceeded 11 or was lower than 3, indicating that the phage could not survive in extremely acidic or alkaline conditions.

The molecular identification of the bacterial strain was obtained by 16S rRNA gene sequence analysis, and then, the homology of the 16S rRNA gene sequence was studied by BLAST search (Fig. S2). The 16S rRNA of the host strain was most similar to CP023019_s (percent of identity at 98.68%), suggesting that it is a sister strain of *Shewanella putrefaciens*.

### Genomic and phylogenetic characterization of vB_SbaS_Y11

**Fig 2:**
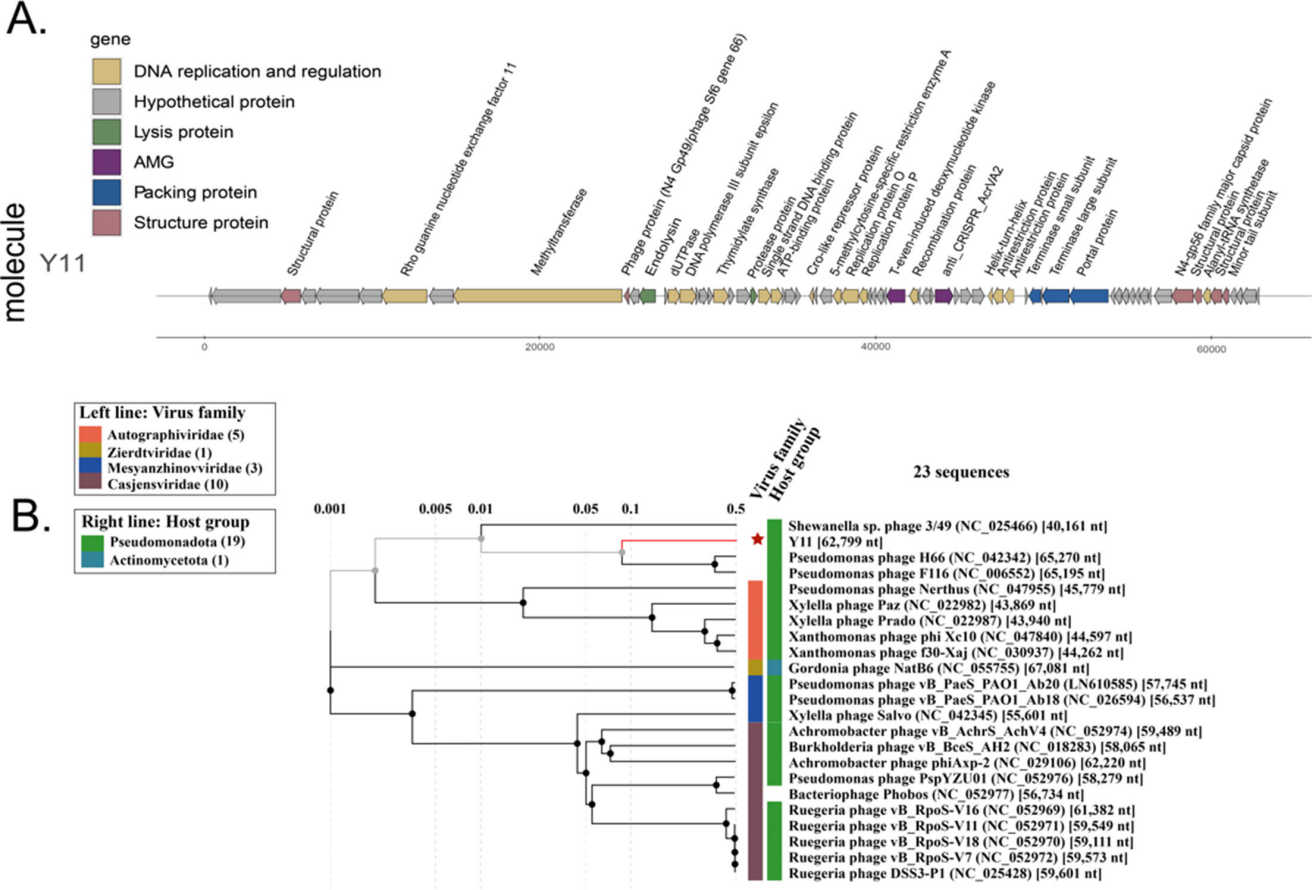
Genome and phylogenetic analysis of *Shewanella* phage vB_SbaS_Y11 with isolated phage genomes. (**A**) Genome map and functional annotation of the predicted proteins of *Shewanella* phage vB_SbaS_Y11. The length of each arrow represents each gene length. The functional genes are divided into six parts with different colors. (**B**) Phylogenetic analysis with other related phages was identified using the genome-wide sequence similarity values computed by tBLASTx. The red star is *Shewanella* phage vB_SbaS_Y11.

According to the sequencing and assembly results, *Shewanella* phage vB_SbaS_Y11 has a linear double-stranded DNA genome of 62,799 bp with a G+C content of 46.9%. No tRNAs were identified in the genome. Protein prediction analysis revealed a total of 71 open reading frames (ORFs) in the genome, with 21 ORFs located on the negative strand and 50 ORFs on the positive strand. Annotated results indicated that 42 ORFs (59.1%) were classified as hypothetical proteins. These ORFs lack retrievable functional annotations under the *E*-value (1*e*−5) threshold (Fig. 2A). On the other hand, the remaining 29 ORFs (40.8%) exhibited high similarity (*E*-value <1^−5^) to known proteins in the database, which have specific functions and were categorized into five modules based on protein function: 15 ORFs were associated with DNA replication and regulation, 1 ORF was related to lysis protein, 3 ORFs were involved in packing proteins, 7 ORFs were associated with structural proteins, and 1 ORF was classified as auxiliary metabolic genes (AMGs).

ORF12 is homologous to an endolysin. Endolysin belongs to a class of cell wall hydrolases encoded by double-stranded DNA phages. The expression of endolysin occurs in the later stages of phage infection and plays a role in the life history strategy of vB_SbaS_Y11, which involves lysing the host cell wall to release daughter phages (19). ORF22 is predicted to encode protease. Polypeptides can be cleaved either chemically or enzymatically. Enzymes that catalyze the hydrolytic cleavage of peptide bonds are called proteases or proteolytic enzymes (20). Depending on the site of peptide bond cleavage, proteolytic enzymes are divided into two groups known as exopeptidase and endopeptidase, which are present in bacteria archaea, certain types of algae, some viruses, and animals. Phage-related ribosomal proteases (Prps) are essential for the assembly and maturation of the ribosome in *Firmicutes*, including the human pathogens *Staphylococcus aureus*, *Streptococcus pneumoniae*, and *Clostridium difficile*. These bacterial proteases cleave off an N-terminal extension of a precursor of ribosomal protein L27, a processing step that is essential for the formation of functional ribosomes. The essential role of Prp in these pathogens has proposed this protease as a potential antibiotic target (21).

Regarding phage packing, ORF51 is predicted to encode the terminase small subunits that specifically bind to concatemeric viral DNA, facilitating the cleavage of DNA by the large subunit and its translocation through the portal protein channel into the procapsid, powered by ATP hydrolysis (22). Additionally, ORF53 act as ATP-driven molecular motors and endonuclease enzymes responsible for cutting the viral genome to initiate and terminate packaging reactions. These processes are essential for the translocation of viral DNA into empty capsids (23, 24).

The structural protein modules predicted in phage vB_SbaS_Y11 included seven ORFs (ORF3, ORF10, ORF63, ORF64, ORF66, and ORF67). ORF63 is predicted to encode N4-gp56 family major capsid protein that presents in phage N4, a double-stranded DNA virus, as well as in many additional lysed phages and pre-integrated phage regions (25). What is more, ORF67 is predicted to encode the minor tail subunit playing an important role in the tail assembly of bacteriophages (26).

DNA replication and regulation accounted for the largest proportion, encompassing 15 identified ORFs. ORF33 is predicted to encode the replication protein P and plays a crucial role in facilitating the replication of the phage chromosome by recruiting key components of the cell’s replication machinery to the virus’s origin. Specifically, it assists in the delivery of one or more *Escherichia coli* DNA helicase molecules into the nucleoprotein structure formed by the lambda O initiator at the origin of lambda replication (27). Interestingly, ORF43 encodes the anti-CRISPR AcrVA2. Bacteria and archaea have developed many systems to defend against potentially harmful mobile genetic elements (MGEs), such as viruses and plasmids. CRISPR and their accompanying Cas genes comprise one of the most prevalent of these systems, occurring in approximately 50% of bacteria and 90% of archaea. CRISPR-Cas systems present a particularly formidable defense because they are adaptive, acquiring specific immunity to segments of foreign DNA after exposure to these elements. To combat these potent systems, MGEs have acquired genes encoding inhibitors of CRISPR-Cas systems, known as Acr proteins also called anti-CRISPR genes. Anti-CRISPR AcrVA2 is one of the Acr found to inhibit the cleavage activity of V-A CRISPR-Cas system effect protein Cas12a. However, the mechanism of AcrVA2 remains unclear (28). ORF47 is predicted to encode the helix-turn-helix (HTH) protein, which consists of an alpha helix of 20 amino acids, acting as a DNA binding motif. It belongs to a large protein family and plays a vital role in maintaining the structural integrity of DNA by binding to the main grooves (29). Shehreen et al. have demonstrated that Acr repression by anti-CRISPR-associated (Aca) proteins is widely conserved in nature (30). The Aca protein, or HTH gene, is distinct from most known anti-CRISPR genes (AcrIE, AcrIF, AcrIC, AcrIIA, and AcrIIC) and can serve as a marker for the presence of anti-CRISPR genes, and most of the findings are consistent with these Acr genes, which are usually located upstream and downstream of HTH genes. To date, 12 Aca genes have been identified in the reported literature. Searching the neighborhood of genomes encoding Aca homologs for potential Acrs and conversely predicting new Aca proteins by identifying Acr downstream genes are approaches that can now be referred to as “guilt-by-association” (31). In the case of phage Y11, this HTH domain gene has also been identified, suggesting that Y11 may be a phage encoding a putative anti-CRISPR gene. ORF48 and ORF49 all predicted to encode the antirestriction proteins, which are similar to the B-form of DNA and work by replacing the DNA in the complex with restriction-modifying enzymes. These proteins function by displacing DNA in complexes that contain restriction-modifying enzymes, thus preventing restriction enzymes from targeting and degrading the viral DNA (32, 33). The deoxyuridine 5′-triphosphate nucleotidohydrolase is also found in several major retroviral groups, shown in several cases to be essential for viral replication (34). ORF9 is predicted to encode methyltransferases and constitutes a diverse group of enzymes that catalyze the transfer of a methyl group from the methyl donor S-adenosyl-l-methionine to various substrates (35).

### Identification of AMGs related to nucleotide metabolism

AMGs are genes integrated within the viral genome that are relevant to their hosts’ physiology. Therefore, analyzing viral AMGs can provide further insight into how viruses potentially impact host metabolism and contribute to microbial community dynamics (36).

In the genome of vB_SbaS_Y11, one AMG was predicted. The first, ORF38, is predicted to encode T2-induced deoxynucleotide kinase, an enzyme that catalyzes the chemical reaction ATP + dGMP (or dTMP) ⇌ ADP + dGDP (or dTDP). The enzyme’s three substrates are ATP, dGMP, and dTMP, while its three products are ADP, dGDP, and dTDP. T2-induced deoxynucleotide kinase belongs to the family of transferases, specifically those that transfer phosphorus-containing groups (phosphotransferases) with a phosphate group as an acceptor (37). Deoxynucleotide kinase in remedial synthesis mainly includes deoxycytidine nucleoside kinase (dCK) and thymine kinase, which are usually more efficient in the remedial synthesis of pyrimidines than purines (38). dCK is the rate-limiting enzyme of dNTP remedial synthesis and is involved in maintaining the stability of the dNTP pool. Also, dCK promotes the phosphorylation of nucleoside analogs such as the chemotherapeutic drugs cytarabine and sapacitabine to inhibit tumor growth (39). dCK phosphorylation and post-translational modifications are essential for its enzymatic activity.

To identify the approximate taxonomic position of the phage vB_SbaS_Y11, a phylogenetic tree based on the viral proteome of whole genomes was established by ViPTree. The results showed that vB_SbaS_Y11 was clustered with the recently isolated *Pseudomonas* virus H66 (NC_042342) and *Pseudomonas* phage F116 (NC_006552) (Fig. 2B). vB_SbaS_Y11 is distant from the other isolated *Shewanella* phages. Although vB_SbaS_Y11 has features belonging to a new group, it is difficult to characterize this new group from these two phages.

### Evolutionary and structural analysis of ORF43

**Fig 3:**
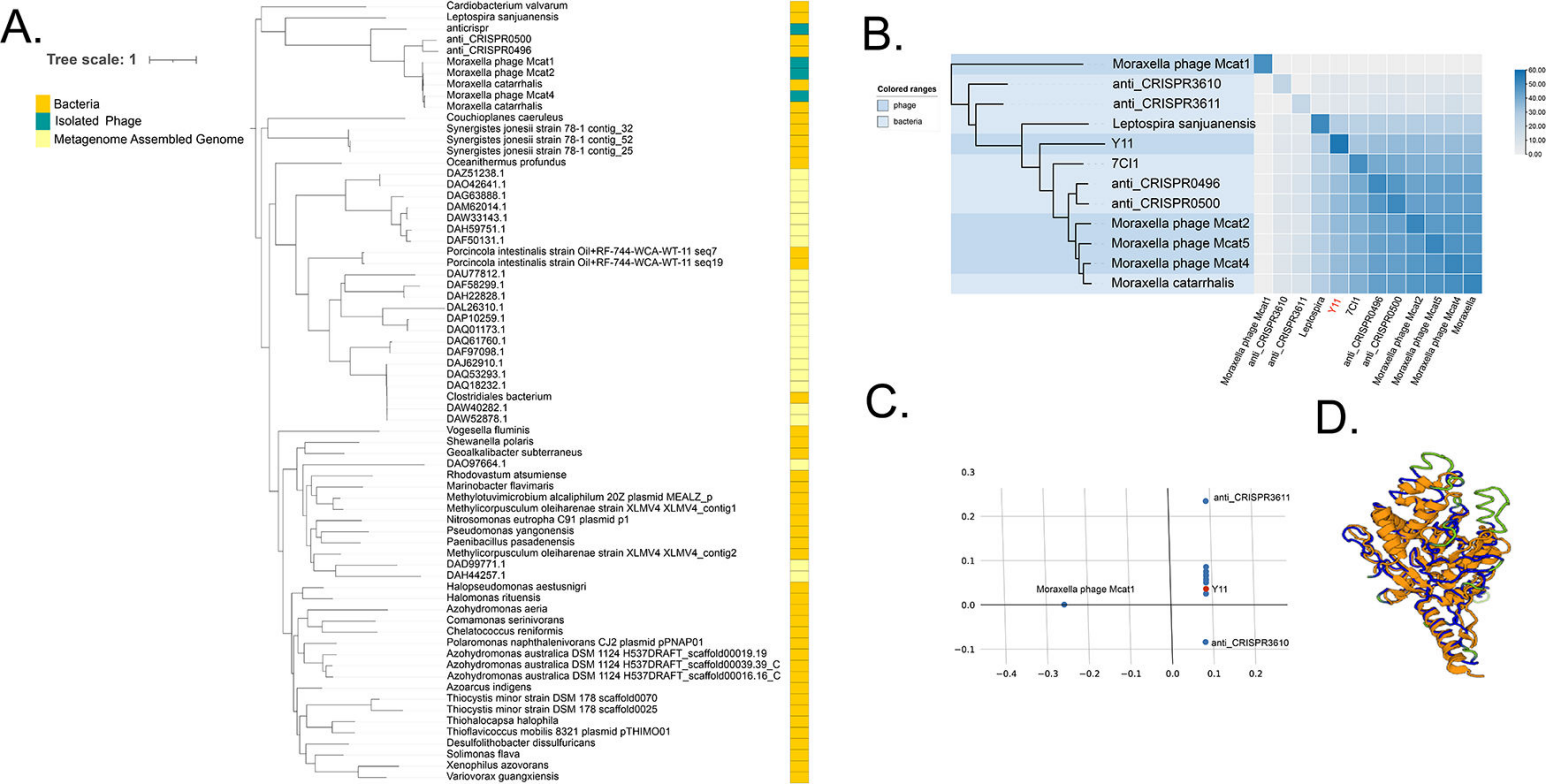
Phylogenetic and structural analyses of ORF43 and associated sequences and proteins. (**A**) A maximum likelihood phylogenetic tree comparing ORF43 with related viruses and bacteria. (**B**) The matrix and clustering dendrogram are based on pairwise *Z*-scores computed using DALI. The data set provides a complete matrix, including actual *Z*-scores. In the dendrogram, different protein kingdoms are highlighted with different background colors. The color bar represents the corresponding *Z*-scores. (**C**) Correspondence analysis of cells and domains computed using DALI. Data points corresponding to ORF43 are positioned relative to each other based on the similarity of their structural neighborhoods. ORF43 is represented in red. (**D**) Structural comparison of ORF43 with the protein from 7CIL.

ORF43 was compared structurally through the DALI server. The proteins of the 11 sequences with the highest similarity to ORF43 were compared to the structural similarity matrix and the corresponding dendrogram analysis. The results revealed that ORF43 had the closer evolutionary position to anti_CRISPR0500 (*Z* = 34.5), anti_CRISPR0496 (*Z* = 33.3), and 7CI1 (*Z* = 30.7) retrieved by DALI search, which was all encoded in the *Moraxella* genus according to PDB searches. The three proteins obtained from ORF43 by BLASTp (*Moraxella* phage Mcat2, *Moraxella* phage Mcat5, and *Moraxella* phage Mcat4) showed high similarity to the three identified anti-CRISPR proteins. The *z*-value-based structural similarity heat map also indicated that ORF43 was correlated with the identified anti-CRISPR proteins (Fig. 3B). Cell structural domain analysis was conducted using DALI, and the corresponding analysis of the data point for ORF43, indicated by red, was determined based on the similarity of its structural neighborhood (Fig. 3C). The results showed that ORF43 was closely located to the known anti-CRISPR AcrVA2. By comparing ORF43 and 7CI1 (anti-CRISPR AcrVA2) in the secondary structure, the results showed that ORF43 and 7CI1 were highly homologous, and structural conservative regions are shown in blue (Fig. 3D). Uppercase means structurally equivalent positions with ORF43. The per-residue confidence score (pLDDT) of the AcrVA2 model presented is above 90 (Fig. S3). The alignment of vB_SbaS_Y11 and verified anti-CRISPR AcrVA2 was performed using MAFFT and visualized with CLC. There were 135 nucleotide positions aligned out of a total of 358, indicating the 37.7% similarity between vB_SbaS_Y11 and verified anti-CRISPR AcrVA2 (Fig. S4). Interestingly, a type I-E CRISPR-Cas system was identified, located at position 56,931–65,565 of the genome of *Shewanella xiamenensis ZYW6*, containing cas2 (ygbF), cas1 (ygbT), casE (cse3, ygcH), casD (cas5e, ygcI), casC (cse4, ygcJ), casB (cse2, ygcK), casA (cse1, ygcL), and cas3 (ygcB) (40). As mentioned earlier, both structural and phylogenetic tree analyses demonstrate that vB_SbaS_Y11 is the first isolated phage that encodes a putative anti-CRISPR AcrVA2 (Fig. S5).

### Three-dimensional structure of AcrVA2

**Fig 4:**
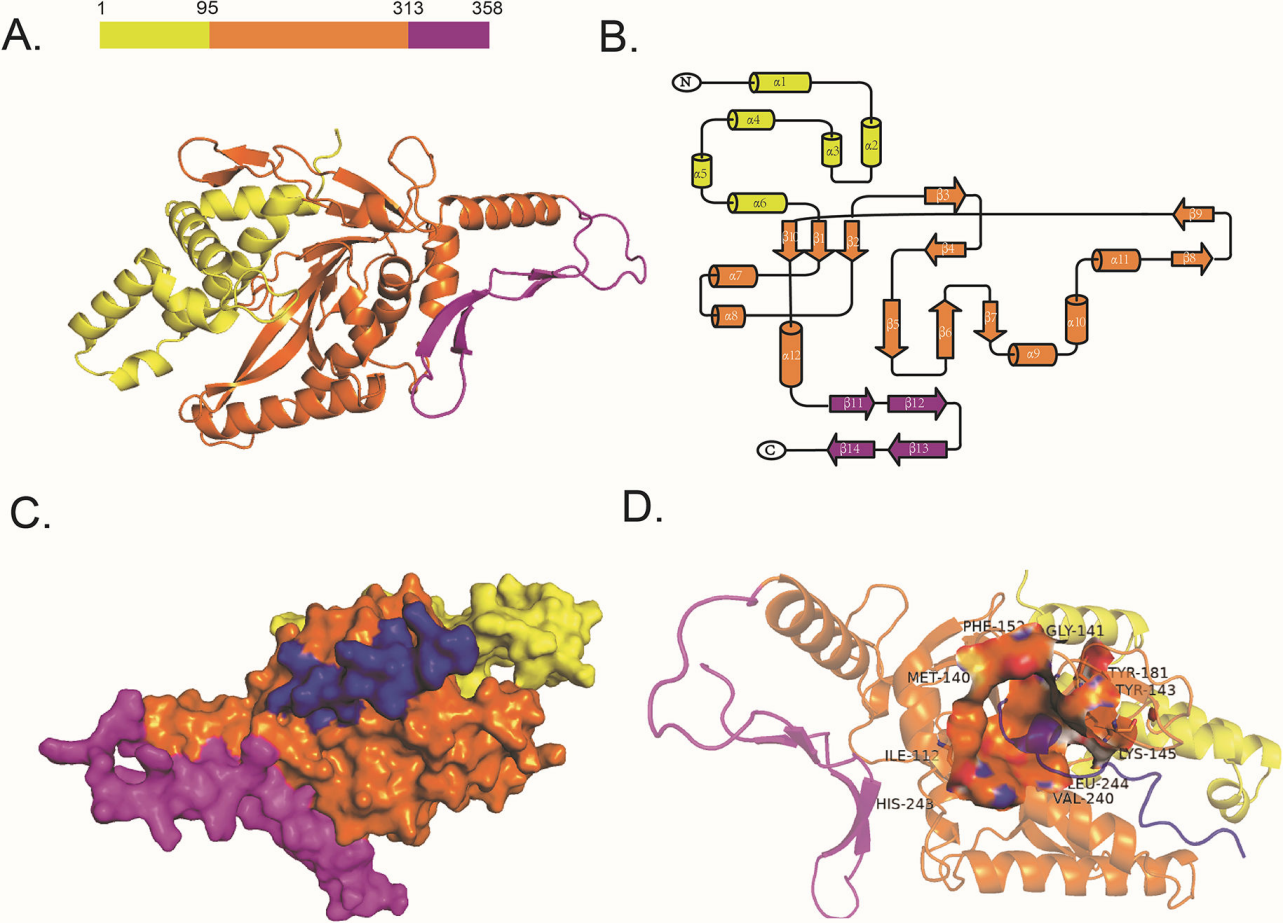
(**A**) Domain organization and overall structure of the AcrVA2. NTD, MID, and CTD domains are colored yellow, orange, and purple. (**B**) Topological diagram of the AcrVA2 structure. (**C**) Surface representation of AcrVA2 in complex with MbCas12a^620-636^. MbCas12a^620-636^ is shown in blue. (**D**) The pocket binding site of MbCas12a^620-636^ and AcrVA2.

The three-dimensional structure of AcrVA2 consists of three structural domains, namely, the N-terminal domain (NTD) spanning residues 1 to 95, the central domain (MID) serving as the main body spanning residues 96 to 313, and the loosely packed C-terminal domain (CTD) spanning residues 314 to 358 (Fig. 4A). The NTD is primarily composed of six α-helices (Fig. 4B). The MID is mainly composed of β-sheet and α-helix structures. The last β-strand (β10) is parallel and adjacent to β1, creating a stable β-sheet structure where the head and tail of the MID meet. This is consistent with the resolution of the AcrVA2 structure (7Cil) predicted by Chen et al. (41). The CTD consists of only four β-strands (β12 and β14, β11 and β13 anti-parallel) (Fig. 4B). Additionally, there is a density loss in residues 313 to 324 and 337 to 342, indicating a higher degree of flexibility in this region. This suggests that the C-terminal of AcrVA2 has some level of flexibility.

### Three-dimensional structure of the MbCas12a^620-636^–ACrVA2 complex

In order to test AcrVA2 and the binding between MbCas12a^620-636^ (41), the ZDOCK protocol of Discovery Studio was used to perform docking between AcrvA2 and significant peptide segments of MbCas12^620-636^. We conducted the docking at 15° rotational sampling density; then, we re-ranked the generated 3,600 docked poses by ZRANK, and the top 2,000 poses were preserved. Then, we filtered the top 2,000 poses by whether the residues of PrankWeb-predicted pocket1 are involved in the protein–protein interface (PPI), with the distance cutoff set to 5 Å. Finally, the filtered four docked poses were refined using the RDOCK protocol. The top 1 docked pose was selected as the final docking result and visualized by PyMOL (Fig. 4C). The results showed that MbCas12a^620-636^ binding at pocket 1 consists of hydrophobic amino acids (Ile-112, Met-140, Gly-141, Phe-152, Val-240, and Leu-244), neutral amino acids (Tyr-143, Tyr-181), and basic amino acids (Lys-145, His-243) (Fig. 4D), suggesting that AcrVA2 protein as an inhibitor of Cas12a may regulate Cas12a activity, but the exact regulatory mechanism needs to be experimentally verified and will be investigated in depth in the future.

### Phylogenetic and comparative genomic analyses

**Fig 5:**
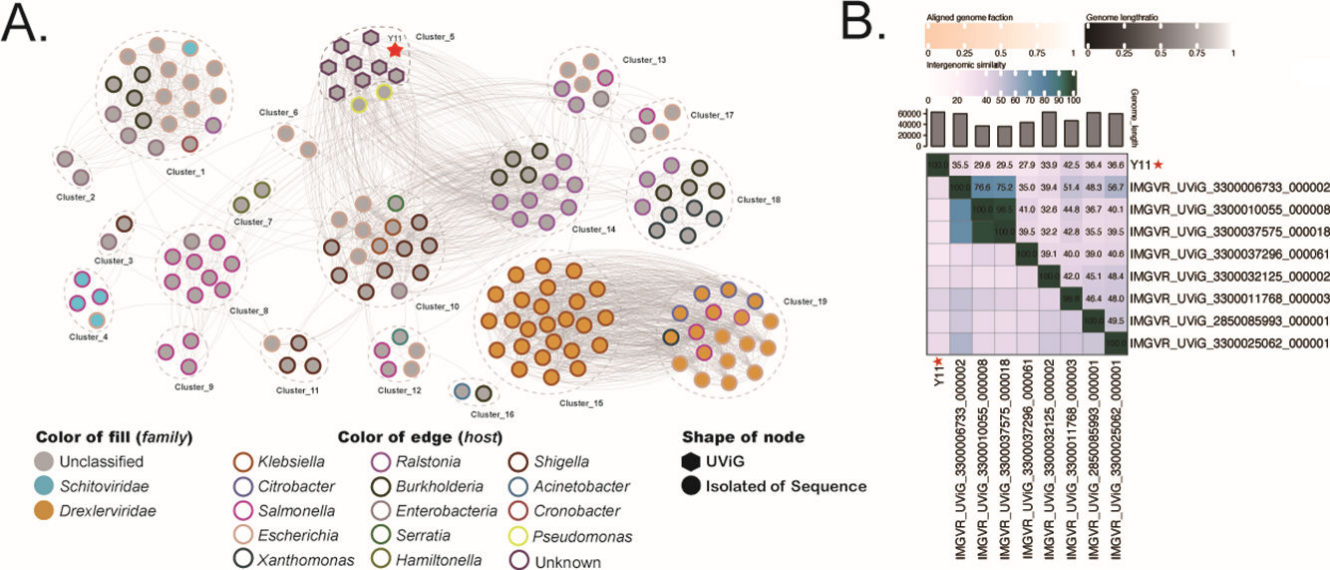
(**A**) Gene content-based viral network among vB_SbaS_Y11 and vB_SbaS_Y11-associated genomes from the GenBank virus database and IMG/VR v4 database. The nodes represent viral genomic sequences. The edges represent the host viral genomic sequences. The isolated viral sequences are indicated by filled circles, and UViGs from IMG/VR v4 are indicated by regular hexagons. Among those, the star represents *Shewanella* phage vB_SbaS_Y11. Viral genomes that belong to different hosts are indicated by different colors. *Shewanella* phage vB_SbaS_Y11 is shown in red. (**B**) Shared gene heat map of vB_SbaS_Y11 and uncultured virus related to vB_SbaS_Y11. In the right half, color coding allows the rapid visualization of phage genome clustering based on intergenic similarity. These numbers represent the similarity values of each genome pair.

In the IMGVR (v.4) database, eight uncultivated virus genomes (UViGs) share more than 30 nucleotide identities with vB_SbaS_Y11, while IMGVR_UVIG_3300011768_000003 has the highest homology in which the percentage of the homology is 42%. Then, vConTACT 2.0 (-pc-inflation 1.2 --link-prop 0.3 --blast-evalue 1e-5) was used to classify 11,008 viruses (GenBank’s 10999 RefSeq, vB_SbaS_Y11 and eight uncultured viral genomic viruses) into genus- or subfamily-level groups. The network diagram shows that vB_SbaS_Y11 was clustered with eight UViGs (Fig. 5A). However, protein clusters (PCs) showed that eight UViGs shared nine proteins with vB_SbaS_Y11. Based on these results, cluster 5 (vB_SbaS_Y11 and eight UViGs) is considered to be a relatively independent cluster from the other identified viral clusters (Fig. 5A). Exploring ANI between different viruses is a common approach for phylogenetic analysis. Cluster 5 was selected together for detailed genomic and comparative genomic analyses in a genome-wide phylogenetic tree. Heat maps of genomic analyses using VIRIDIC showed that their genome lengths are basically between 40 and 60 kb. vB_SbaS_Y11 had gene-sharing rates with other genomes ranging from 7.0 to 42.5 (Fig. 5B).

The presence and absence of conserved genes and specific protein sequences can serve as crucial evidence for establishing a potential new family virus. Genomes associated with vB_SbaS_Y11, obtained from the NCBI virus database and the IMG/VR v4 data set, were subjected to protein clustering analysis. The leftmost section represents various genomes related to vB_SbaS_Y11, with four different colors representing distinct viral clusters (VCs), each containing different PCs. vB_SbaS_Y11 is indicated by a red star, solid lines denote the presence of protein clusters, and dashed lines indicate their absence.

**Fig 6:**
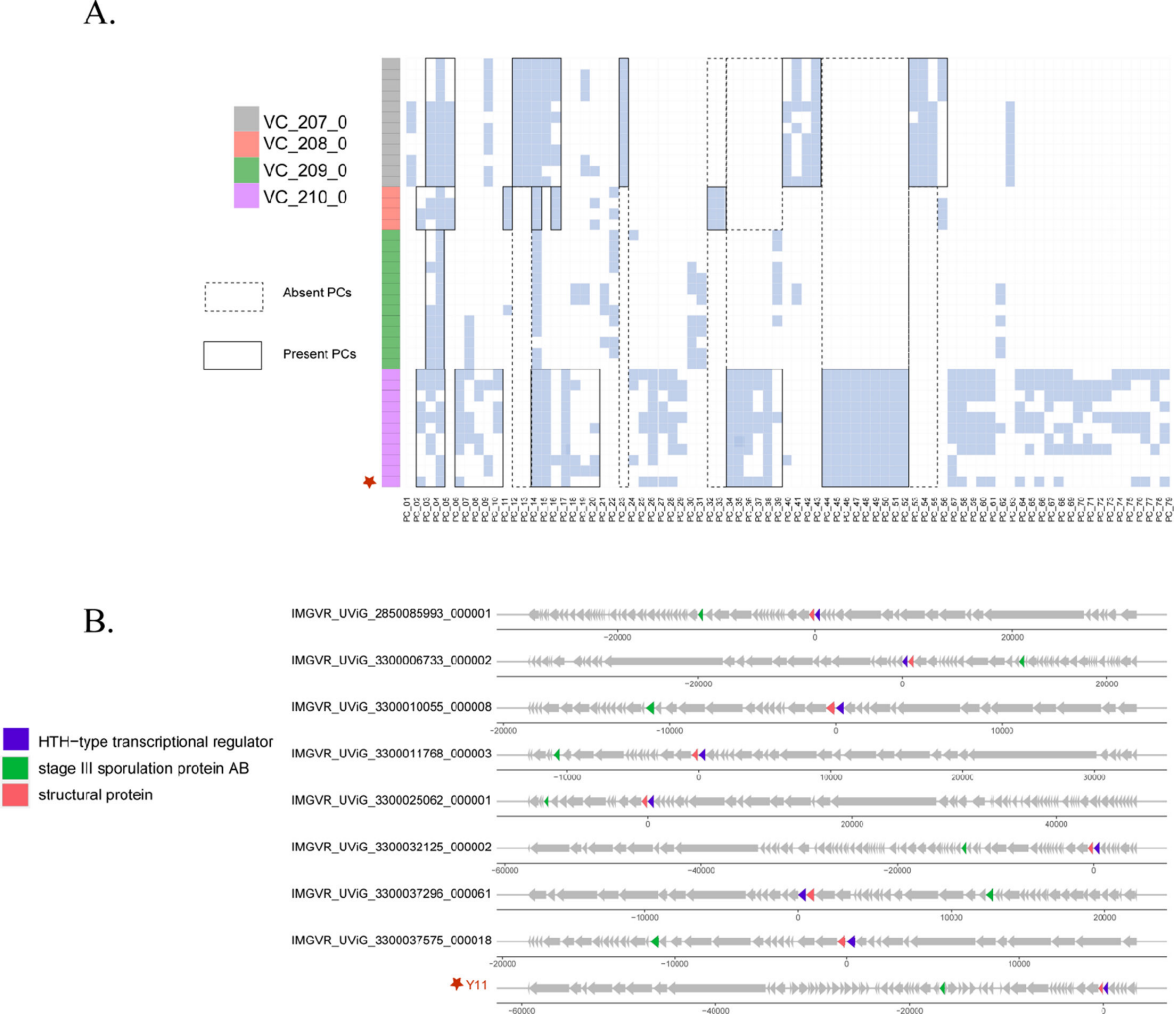
(**A**) Protein cluster analysis between vB_SbaS_Y11 and vB_SbaS_Y11-associated genomes from the GenBank virus database and IMG/VR v4 data set. Blocks on the left represent different genomes, and different colors represent different VCs. The solid black border indicates PCs presented, and the dotted black border indicates PCs absent. The blue squares indicate that a certain PC is present in a specific VC, while the white squares denote its absence. vB_SbaS_Y11 is labeled as a red star. (**B**) The gene map of conserved proteins between vB_SbaS_Y11 and other phages. The conserved proteins are shown by different colors.

The results revealed that the proteins present in the VC_210_0 viral cluster, including vB_SbaS_Y11, exhibited high similarity within the cluster and showed greater divergence from the proteins present in other viral clusters (Fig. 6A). VC_207_0 consisted of at least one protein from PC3-PC5, two proteins from PC40-PC43 and PC53-56, and three proteins from PC12-PC16, all of which included PC23. VC_208_0 contained PC11, PC14, PC16, PC32, and PC33 and at least one protein from PC2-PC5 but did not include PC12, PC13, PC23, PC40-PC43, and PC53-PC55. VC_209_0 only contained at least PC3 and PC4, with numerous absences in other PCs. vB_SbaS_Y11 was clustered into VC_210_0, which included one protein from PC2-4, two proteins from PC6-PC10, and three proteins from PC14-20 and PC34-PC39, but did not detect PC44-PC52 in other sequences, demonstrating the specificity of the *Shewanella* phage vB_SbaS_Y11 genomes.

The nine core genes are mainly distributed in three modules. Rho guanine nucleotide exchange factor 11 (ORF7), stage III sporulation protein AB (ORF41), and HTH-type transcriptional regulator (ORF65) are located in the DNA nucleotide metabolism module. ORF2, ORF5, and ORF6 are distributed in the hypothetical module. Structural proteins (ORF43, ORF64, and ORF66) are distributed in the structural module. In addition, nine core genes are clustered in the regions preceding and following vB_SbaS_Y11. Subsequent analysis utilizing BLASTp to search for these nine core proteins in the nonredundant (NR) isolated database revealed that three core genes possess these nine protein clusters and are exclusively present in cluster 5. Consequently, these three proteins can be designated as conserved proteins (Fig. 6B). In order to further investigate the phylogenetic characteristics of cluster 5, BLASTp was used to compare three conserved proteins (ORF41, ORF64, and ORF65) in the Nonredundant Protein Sequence Database, limiting the search scope to the virus (Table S1). The results showed that the viruses in cluster 5 all formed a single branch on the phylogenetic tree and similar to the results of the whole gene protein tree, indicating that they also have monophyletic traits in phylogeny (Fig. S6). A phylogenetic tree based on the terminal enzyme large subunits of all viruses related to vB_SbaS_Y11 was constructed. vB_SbaS_Y11 formed a single branch with eight uncultured viruses, indicating their phylogenetic monophyly (Fig. S7). Based on these findings, it is suggested that cluster 5 represents a novel potential family, which is named here as *Ranviridae*.

### Ecological distribution of vB_SbaS_Y11 in the ocean

**Fig 7:**
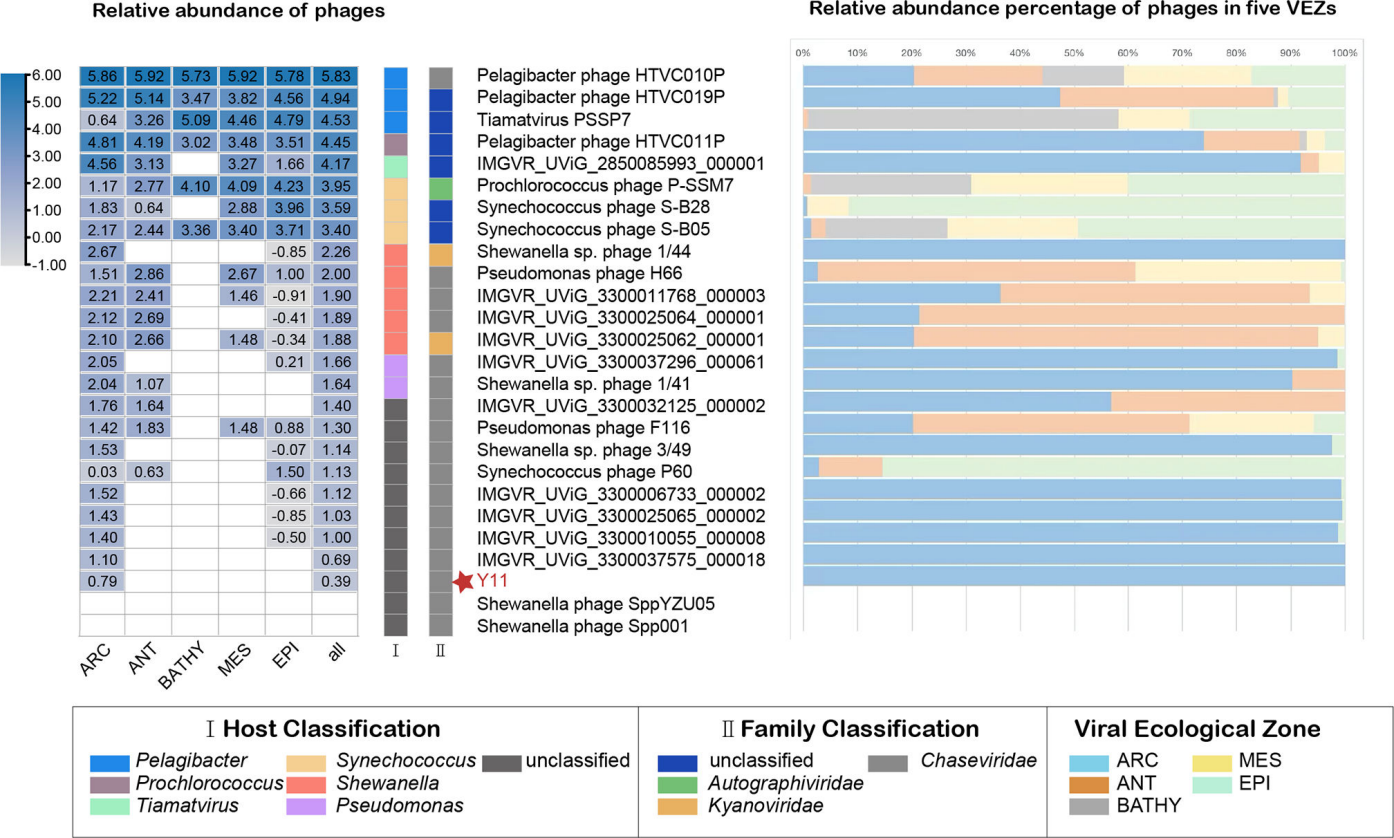
Relative abundance of the *Shewanella* phage vB_SbaS_Y11 and other important phages in the 154 virome data set of GOV2.0. Relative abundance represents the value calculated for Tpm (transcripts read per million mappings) and CoverM (v0.3.1). Values are normalized according to the number of databases. The bar on the right shows the VEZ. The five ocean VEZs are ARC, ANT, BATHY, EPI, and MES. The result of the heat map on the left is log10 convert. *Pelagibacter* (SAR11) phages, *cyanophages*, Puniceispirillum (SAR116) phage HMO-2011, and isolated and related bacteriophages of *Shewanella* phage vB_SbaS_Y11 were used as references.

To investigate the biogeographical distribution of vB_SbaS_Y11, we examined its presence in 154 viral metagenomes obtained from five different virus ecological zones (VEZs) in the Global Ocean Viromes (GOV2.0) data set, including the Arctic (ARC), Antarctic (ANT), temperate and tropical epipelagic (EPI), temperate and tropical mesopelagic (MES), and bathypelagic (BATHY) VEZs. Five *Shewanella* phages with similar genome sizes to vB_SbaS_Y11 and high abundance in the oceans were selected as references for the abundance assessment with vB_SbaS_Y11 in the above database. In the five VEZs, the relative abundance of vB_SbaS_Y11 and the other five *Shewanella* phages in ANT and MES was generally low (Fig. 7). vB_SbaS_Y11 was only detected in the ARC and was not found in other VEZs. Furthermore, IMGVR_UViG_2850085993_000001 was primarily detected in the MES, BATHY, ANT, and ARC regions, with the highest relative abundance detected in the ARC. On the other hand, Shewanella phages SppYZU05 and Spp001 were not detected in any of the VEZs in the GOV2.0 data set (Fig. 7). Currently, in the NCBI virus database, of the 21 complete genomes of phages isolated from *Shewanella*, most of them were isolated from sea ice, mines, seafood, lakes, and coasts, which also suggests that *Shewanella* are widely distributed in the marine environment. In contrast, there are fewer phages isolated from marine environments; even vB_SbaS_Y11 was isolated from seafood off the coast of Qingdao, possibly representing a preference for vB_SbaS_Y11 to be distributed near the shore rather than miles offshore in the ocean. This may be the reason why the abundance of vB_SbaS_Y11 is low in GOV. However, it may play an important role in the ecological remediation of heavy metal contaminants in metal-contaminated environments (18), thereby contributing to the stability of aquatic environmental resilience.

## MATERIALS AND METHODS

### Isolation and purification of phage and host

Host strain *Shewanella* and phage vB_SbaS_Y11 were isolated from the sewage of the seafood market in Qingdao, China (36°4′9″N, 120°20′13″E). The obtained samples were diluted to 10^−8^, and 200 µL of the diluted sample was taken and inoculated onto the 2216E solid medium using the spread plate method. The medium was inverted in the incubator at 28°C, and the above purification steps were repeated more than three times. The purified host *Shewanella* 16s rRNA was amplified using PCR and sequenced (42).

A double-layer method was used to isolate the phage (43). The sewage samples were filtered through the 0.22 µm membrane (microporous) to remove bacteria. We took 200 µL of filtered sewage and 200 µL from the logarithmic growth period of the host, incubated it for 25 minutes, and added 3.5 mL of the semi-solid 2216E medium for overnight incubation, then poked a single phage plaque and stored it in SM solution. The above steps were repeated three times to obtain the purified phage stock solution. The purified phage was expanded and cultured to 500 mL and then filtered using a 0.22 µm membrane filter (microporous). The phage culture was concentrated to 3 mL using an ultrafiltration tube (microporous Amicon Ultra-15) at 5,000 *g* (44). The purified phage was preserved in SM buffer at 4°C for further processing.

### Transmission electron microscopy

The morphological characteristics of vB_SbaS_Y11 were observed through TEM. Firstly, 20 µL of the concentrated phage solution was dispensed onto a copper grid and then let stand for 15 minutes without light. The negative stain was performed with phosphotungstic acid (2 wt%, pH 7.5) for 5 minutes (45). The JEOL model JEM-1200EX TEM, containing the GATAN INCCCD image transfer system, was utilized to capture clear electron micrographs at 100 kV. Subsequently, electron micrographs were used to estimate the phage dimensions.

### One-step growth assays

The bacterial and purified phage solutions were mixed with multiplicity of infection (MOI) of 0.1 and incubated for 20 minutes at 28°C. The nonabsorbed phage was removed by centrifugation, and the remaining unabsorbed phages were removed by using a fresh 2216E liquid medium to wash the precipitate three times. The samples were taken at different intervals: every 5 minutes for the first 30 minutes and every 10 minutes from 30 to 90 minutes. The viral titers were determined using the double-layer method, and the growth curve was plotted by counting the number of plaques at various time points (46). To ensure the accuracy of the results, the experiment was repeated three times in parallel.

### Thermal and pH stability assays

The 500-µL phage solution (initial titer ~10^9^ PFU/mL) used HCl and NaOH to adjust the pH of the phage solution. Eleven parts of the virus solution were prepared with different pH values of 2 to 12, respectively. The phage solution was placed for 2 hours (47). The 200-µL phage solution treated with different pHs was mixed with 200 µL of the host bacterial solution, which was in the logarithmic growth stage, with a concentration of 10^8^ CFU/mL.

The logarithmic growth phase host bacterial solution was mixed with the treated phage solution, and the mixture was incubated at room temperature for 25 minutes. Then, the semi-solid culture medium was added to the mixture and poured into the solidified culture medium to form a double-layer plate, which was incubated overnight at a constant temperature to determine the survival rate of the phage. Plaques were counted, and the phage growth curve at different temperatures was plotted (44, 47). To ensure accuracy, three sets of parallel experiments were conducted.

### Phage DNA extraction, sequencing, and annotation

The extraction of phage genomic DNA used a viral DNA kit (OMEGA), and the detection of nucleic acids was by electrophoresis (46). Purified phage genomic DNA was sequenced by Lingen (Shanghai, China) using the Illumina Miseq 2× 300 paired-end method. Raw data were processed with soapnut (v2.0.5) software to obtain high-quality clean reads, which were used for Illumina NovaSeq 6000 sequencing. BWA (v0.7.17, default parameter: mem–K30) software was subsequently applied to remove the influence of the host sequence. Raw paired-end reads were trimmed and quality-controlled by trimmatic (ILLUMINACLIP: adapters.fa:2:30:10 SLIDINGWINDOW:4:15MINLEN:75) (48). ABySS (http://www.bcgsc.ca/platform/bioinfo/software/abyss) was used to stitch multiple Kmer parameters on the optimized sequence to obtain the optimal assembly result (49). The single-base polymorphism and the remaining local inner gaps were corrected for the final assembly and further analysis by GapCloser (https://sourceforge.net/projects/soapdenovo2/files/GapCloser/) (50).

### Genome annotation and comparative genomic analysis

Rapid Annotation using Subsystem Technology (RAST, http://rast.nmpdr.org/) was used to predict the ORFs. The annotation used BLASTp against the nonredundant BLASTp (http://blast.ncbi.nlm.nih.gov/), HHpred against the PDB_mmCIF30_18_Jun, Pfam-A_v35, UniProt-SwissProt-viral70_3_Nov_2021, and NCBI_Conserved_Domains (CD)_v3.19 (https://toolkit.tuebingen.mpg.de/hhpred). An HMM search against the Pfam database was performed on their web server (https://pfam.xfam.org) with default parameters used to detect conserved domains in each ORF with the *E*-value <1^−5^. The annotation information generated by different databases was manually checked based on the *E*-value and bitscore. The tRNAscan-SE (http://lowelab.ucsc.edu/tRNAscan-SE/) was used to identify tRNA (51). Genome mapping was performed using Rstudio. The whole genome phylogenetic tree based on the amino acid sequence of phage vB_SbaS_Y11 and comparative genomic analysis was constructed using ViPTree v3.1 (https://www.genome.jp/viptree) (52). Phageterm was used to identify the phage termini (53).

### Structural proteome and phylogenetic tree of ORF43

The tertiary structure of ORF43 encoding protein was predicted by ColabFold (54) and visualized with PyMOL (55). ORF43 BLASTp was against the NR bacterial, archaeal, and viral database (*E*-value ≤1*e*−5, coverage ≥30, and amino acid identity ≥30). The aligned sequences were then used to construct a phylogenetic tree and visualized using MAFFT and ITOL. Protein structure-based searches were performed using the DALI server (http://ekhidna.biocenter.helsinki.fi/dali/) (56). Structural similarities between cellular and viral proteins were evaluated based on the DALI *Z* score, which is a measure of the quality of the structural alignment. *Z* scores above 2, i.e., 2 SDs above expected, are usually considered significant. The relevance of the matches was evaluated further by a visual inspection of structural alignments between the compared proteins. The active site of ORF43 was predicted by PrankWeb (https://prankweb.cz/). Discovery Studio (57) was used for peptide docking and visualized by PyMOL.

### Network analysis

The default parameters of ClusterONE were used to define and identify the VCs. ClusterONE is an algorithm for identifying protein complexes in protein interaction networks. A density-based clustering algorithm that can find densely connected protein subgraphs in large-scale protein interaction networks and identify them as potential protein complexes was used. The specific inputs are the protein interactions obtained by BLASTp and their weights, which are selected from the algorithm in the vContact2 tool. The clustering analysis was performed using NCBI-RefSeq (standardized, validated database of genomes, transcripts, and protein sequences provided by NCBI) and phages associated with vB_SbaS_Y11. To search for viral sequences homologous to vB_SbaS_Y11 in NCBI and IMG/VR v4 (54), diamond BLASTp is used to calculate the sequence similarity between proteins, in particular for predicting potential pairings of protein interactions (PPIs) (*E*-value ≤1*e*−5, coverage ≥30, and amino acid identity ≥30). Viral sequences sharing more than 30% ORFs of vB_SbaS_Y11 were used as reference for vConTACT. The network analysis that used vConTACT 2.0 was performed based on the ICTV classification data set to cluster and provide a taxonomy of the sequencing data (58). The VIRIDIC analysis included eight UVIGs belonging to the same VC in vConTACT, two *Pseudomonas* phages that have been isolated were used for nucleotide identity analysis, and Gephi 0.9.2 was used for network visualization (59).

### Conserved protein analysis

A heat map analysis of the shared genes between vB_SbaS_Y11 and related VC protein sequences generated by the Pheatmap package in R was performed. Shared genes by BLASTp were against the NR database (*E*-value ≤1*e*−5, coverage ≥30, and amino acid identity ≥30) and three conserved protein gene map mappings generated by the gggenes package in R (47).

### Ecological distribution in the GOV2.0 (ocean)

The relative abundance of vB_SbaS_Y11 is expressed as tpm (per million transcripts) and calculated by CoverM (v0.3.1) (parameters: –min-read-percent-identity 0.95–minread-aligned-percent 0.75) (60). The five marine VEZs defined by the GOV2.0 data set are ANT, ARC, BATHY, EPI, and MES (61). In addition, *Pelagiphage* HTVC010P, *Prochlorococcus* P-SSP7, *Pelagiphage* HTVC019P, and *Pelagiphage* HTVC011P are significantly representative in the oceans and have other 10 viruses in the same cluster and five viruses with *Shewanella* phage complete genomes in the GeneBank as the references (62). After adding the relative abundance of viruses in five VEZs, it was converted into log10, and the subsequent results were visualized by Tbtool software and R packages.

### Conclusion

Bacteriophages play pivotal roles in the ecological and evolutionary success of microbial communities. However, our knowledge regarding the *Shewanella* phages, which significantly impact the structure and dynamics of *Shewanella* communities, remains limited. By isolating and characterizing a novel phage vB_SbaS_Y11, we have expanded our understanding of the genome, diversity, evolution, and phage–host interactions of *Shewanella* phages. In this study, *Shewanella* phage vB_SbaS_Y11, which carries a putative anti-CRISPR AcrVAR2 gene, is isolated and classified and represents a potential new family, *Ranviridae*, comprising eight UViGs. It is worth noting that future research focusing on horizontal gene transfer between different species and the global distribution of *Shewanella* will be crucial to enhancing our comprehension of the genomic characteristics and physiological and genetic diversity, as well as the ecological distribution of *Shewanella* phages in marine ecosystems. Further isolation and analysis of additional *Shewanella* phages are required to elucidate the intricate interactions between *Shewanella* and their corresponding phages encoding anti-CRISPR AcrVAR2. We provide a structural basis for further understanding the AcrVAR2 protein inhibition of Cas12a activity and provide a basis for the development and application of inhibitors of Cas12a activity.

## Data Availability

The genome sequence of phage vB_SbaS_Y11 has been deposited in GenBank under accession number OQ927986. The 16S rRNA sequence of the host also has been deposited in NCBI under accession number OR016665.

## SUPPLEMENTAL MATERIAL

Supplemental material spectrum.03367-23-s0001.doc Fig. S1 to S7; Table S1.

ASM does not own the copyrights to Supplemental Material that may be linked to, or accessed through, an article. The authors have granted ASM a non-exclusive, world-wide license to publish the Supplemental Material files. Please contact the corresponding author directly for reuse.
