# Supplementary material for: Shewanella phage encoding a putative anti-CRISPR-like gene represents a novel potential viral family
Source: Microbiol Spectr. 2024 Jan 12;12(2):e03367-23. doi: 10.1128/spectrum.03367-23 (PMC10846135; doi:10.1128/spectrum.03367-23)
Supplement: Supplemental material — Fig. S1 to S7; Table S1. [file spectrum.03367-23-s0001.doc]

**SUPPLEMENTAL MATERIALS**

**Fig. S1** The phage morphology of Y11.

**Fig. S2** The phylogenic tree based on the 16S rRNA gene of *Shewanella kR11* and other 50 reference.

**Fig. S3** The per-residue confidence score (pLDDT) of the ORF43 model.

**Fig. S4** Conservative positions on verfied anticrispr_AcrVA2 and Y11 were indicated.

**Fig. S5** The conservative positions were compared in ORF43 and related virus and bacteria.

**Fig. S6** The phylogenetic tree of three conserved proteins (ORF 41, ORF 64, ORF65) were compared.

**Fig. S7** The phylogenic tree of Y11 based on terminase large subunits.

**Table S1** Three core proteins blast against Nr database.

**Fig. S1**


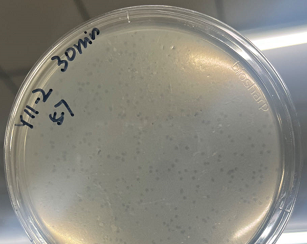


**Fig. S2**


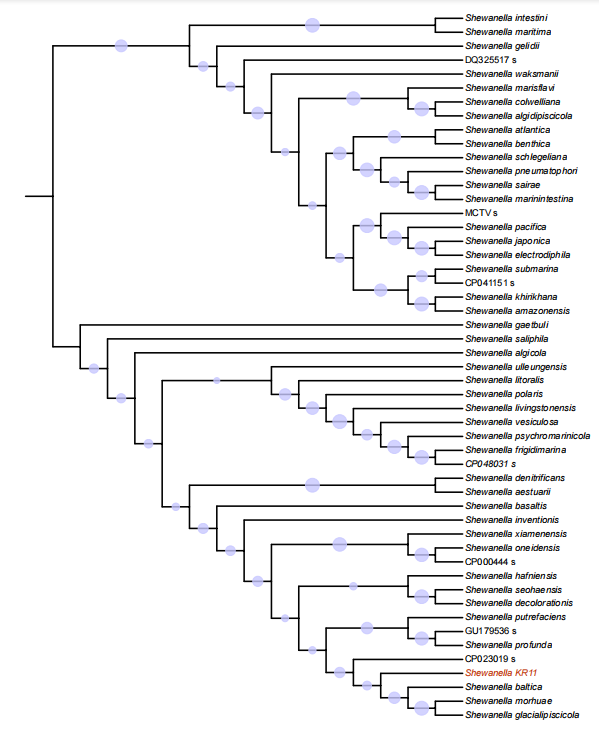


**Fig. S3**


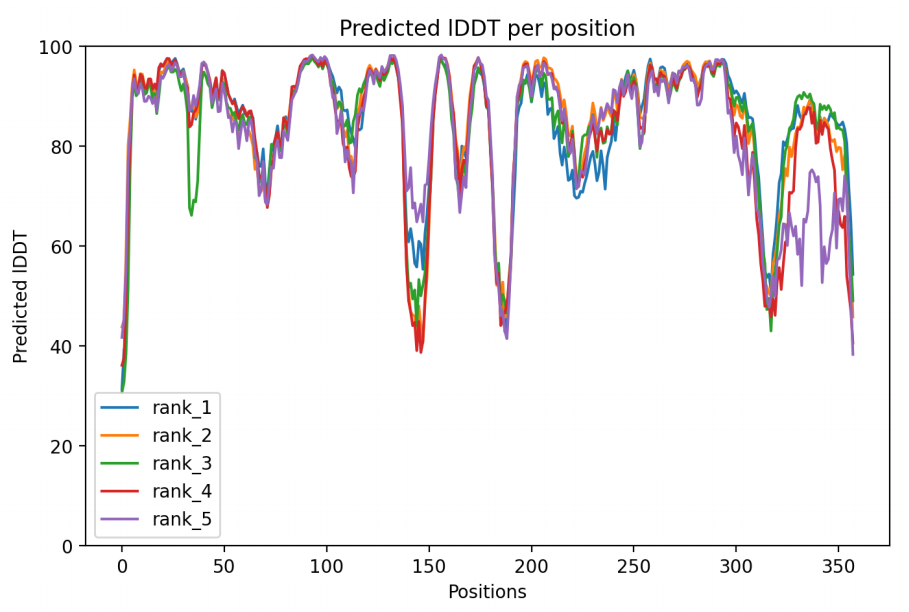


**Fig. S4**


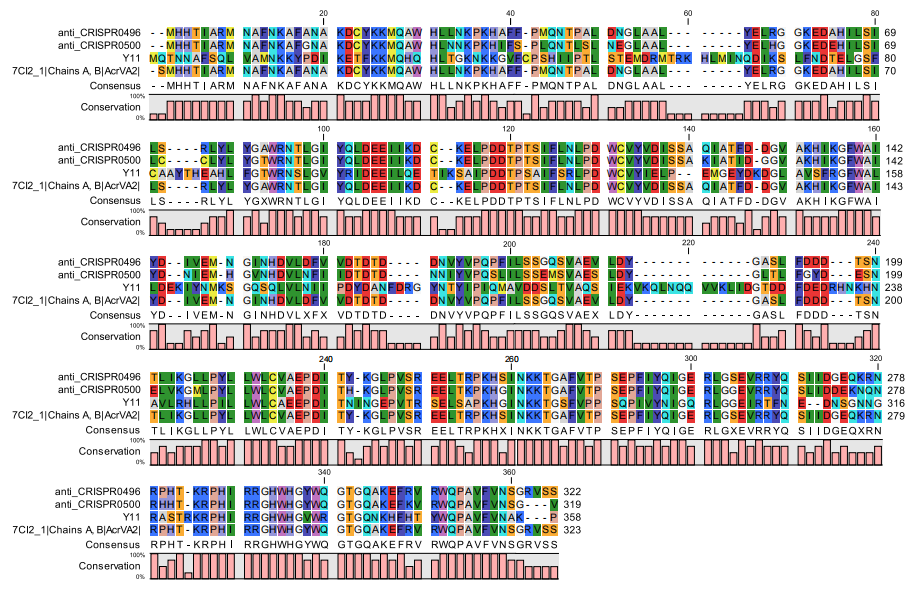


**Fig. S5**


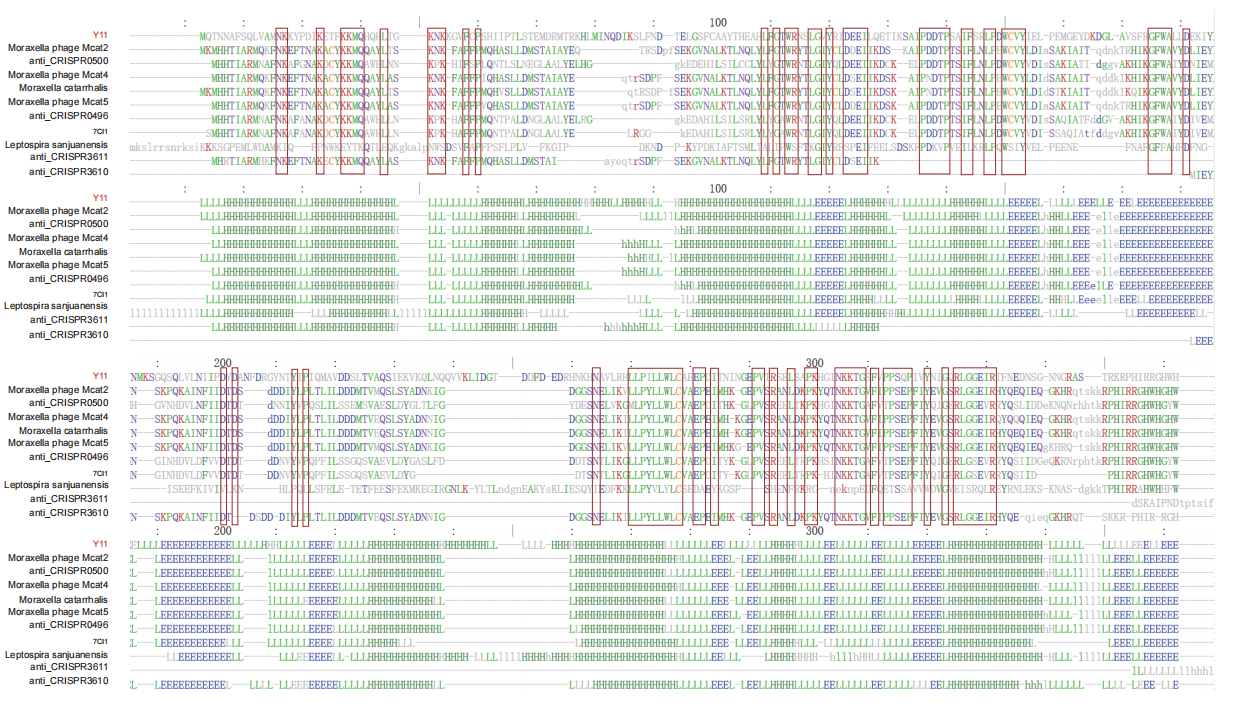


**Fig. S6**


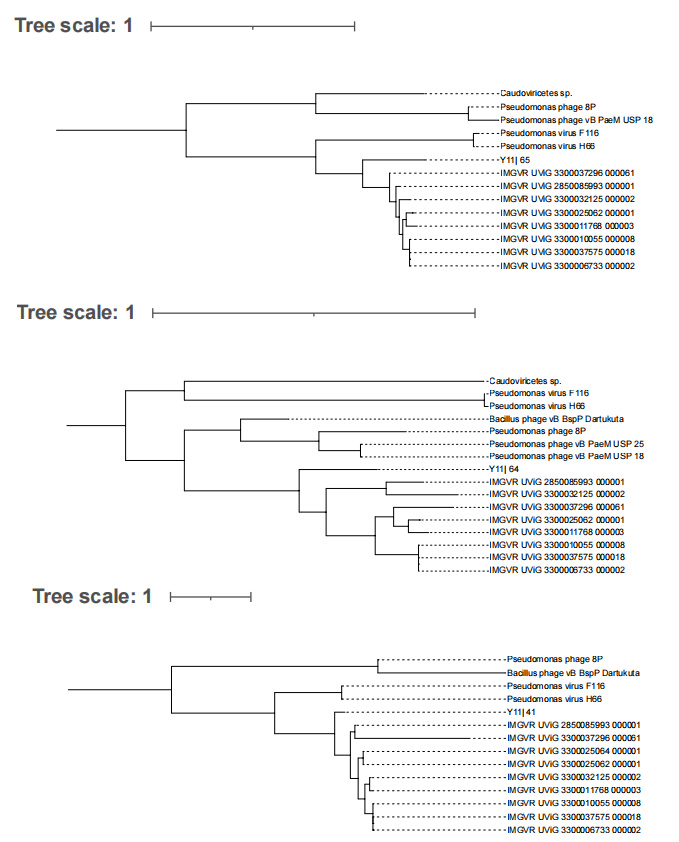


**Fig. S7**


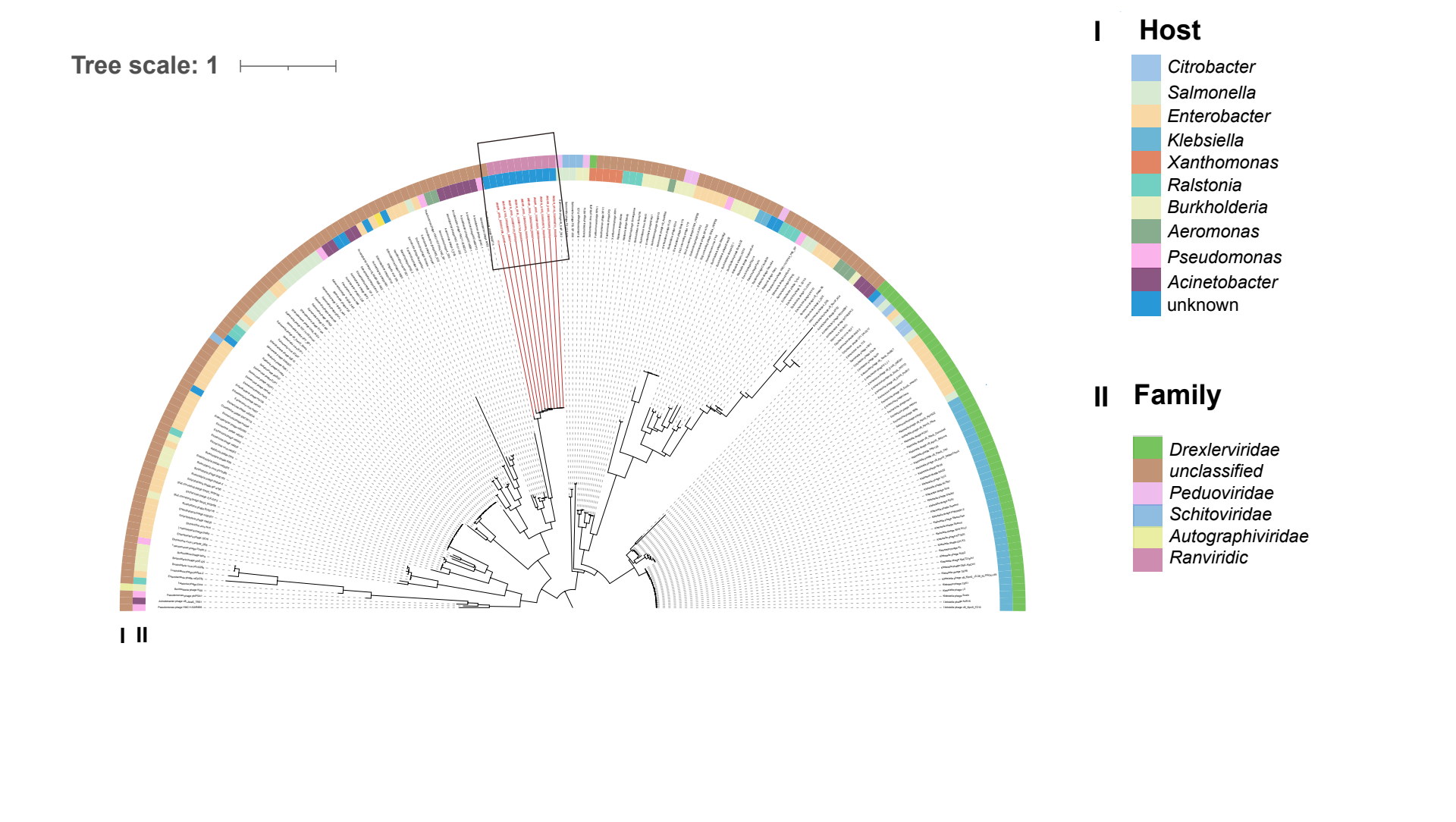


**Table S1 Three core proteins blast against Nr database.**

| qseqid | sseqid | evalue | bitscore | taxid | classification |
| --- | --- | --- | --- | --- | --- |
| Y11_41 | QIW89242.1 | 3.29E-47 | 161 | 2723907 | Viruses |
| Y11_41 | QLI49437.1 | 2.68E-46 | 159 | 2743937 | Viruses |
| Y11_41 | WP_061362845.1 | 1.27E-34 | 129 | 287 | cellular organisms |
| Y11_41 | WP_004354941.1 | 1.27E-34 | 129 | 287 | cellular organisms |
| Y11_41 | WP_033970916.1 | 1.31E-34 | 129 | 287 | cellular organisms |
| Y11_41 | WP_004349189.1 | 1.31E-34 | 129 | 287 | cellular organisms |
| Y11_41 | WP_019486228.1 | 1.31E-34 | 129 | 286 | cellular organisms |
| Y11_41 | WP_033976674.1 | 1.31E-34 | 129 | 136841 | cellular organisms |
| Y11_41 | WP_015975447.1 | 7.47E-34 | 127 | 286 | cellular organisms |
| Y11_41 | DAY12550.1 | 4.85E-17 | 84 | 2832643 | Viruses |
| Y11_64 | QXN70896.1 | 1.27E-54 | 180 | 2836117 | Viruses |
| Y11_64 | QIW89263.1 | 3.57E-48 | 163 | 2723907 | Viruses |
| Y11_64 | QLI49441.1 | 5.67E-47 | 160 | 2743937 | Viruses |
| Y11_64 | QLI49507.1 | 3.39E-46 | 158 | 2743939 | Viruses |
| Y11_64 | DAY12547.1 | 1.51E-34 | 129 | 2832643 | Viruses |
| Y11_65 | QIW89264.1 | 5.16E-18 | 89.4 | 2723907 | Viruses |
| Y11_65 | QXN70897.1 | 1.28E-14 | 80.1 | 2836117 | Viruses |
| Y11_65 | WP_086236398.1 | 3.55E-09 | 65.5 | 287 | cellular organisms |
| Y11_65 | WP_058174921.1 | 3.55E-09 | 65.5 | 287 | cellular organisms |
| Y11_65 | WP_034017968.1 | 3.55E-09 | 65.5 | 287 | cellular organisms |
| Y11_65 | WP_086352069.1 | 6.73E-09 | 64.7 | 287 | cellular organisms |
| Y11_65 | WP_015975450.1 | 2.41E-08 | 63.2 | 287 | cellular organisms |
| Y11_65 | WP_004354927.1 | 2.41E-08 | 63.2 | 286 | cellular organisms |
| Y11_65 | WP_071580243.1 | 2.41E-08 | 63.2 | 136841 | cellular organisms |
